# A comparison of indirect and direct targeted STN DBS in the treatment of Parkinson’s disease—surgical method and clinical outcome over 15-year timespan

**DOI:** 10.1007/s00701-020-04269-x

**Published:** 2020-02-26

**Authors:** Maija Johanna Lahtinen, Tarja Helena Haapaniemi, Mikko Tapio Kauppinen, Niina Salokorpi, Esa Raimo Heikkinen, Jani Petteri Katisko

**Affiliations:** 1 Department of Neurosurgery, Oulu University Hospital, Kajaanintie 50, FI-90220 Oulu, Finland; 2 Oulu Research Group of Advanced Surgical Technologies and Physics, Medical Research Center Oulu (MRC Oulu), Oulu University Hospital and University of Oulu, Oulu, Finland; 3 Research Unit of Clinical Neuroscience, University of Oulu, Oulu, Finland; 4 Department of Neurology, Oulu University Hospital, Oulu, Finland

**Keywords:** Deep brain stimulation, Parkinson’s disease, Subthalamic nucleus, Magnetic resonance imaging, Targeting

## Abstract

**Background:**

Deep brain stimulation (DBS) in the subthalamic nucleus (STN) is used in advanced Parkinson’s disease (PD) for reducing motor fluctuations and the side effects of antiparkinsonian medication (APM). The development of neuroimaging has enabled the direct targeting of the STN. The aim of this study is to evaluate the outcome in patients with PD using STN DBS when changing from atlas-based indirect targeting method (iTM) to direct MRI-based targeting (dTM) assuming dTM is superior.

**Methods:**

Twenty-five consecutive PD patients underwent dTM STN DBS surgery from 2014 to 2017 with follow-up for 1 year. The neuroimaging, surgical method, outcome in Unified Parkinson’s Disease Rating Scale (UPDRS) scores, and reduction of APM are described and compared with the results of an earlier iTM STN DBS study.

**Results:**

Twelve months after a dTM STN DBS, significant improvement (*p* < 0.001) was seen in six out of seven parameters of UPDRS when patients had medication (medON) and stimulation (stimON). The activities of daily living (UPDRSII) and motor scores (UPDRSIII) improved by 41% and 62%, respectively. Dyskinesias and fluctuations were both reduced by 81%. In dTM STN DBS group, the levodopa equivalent dose (LED) and the total daily levodopa equivalent dose (LEDD) were significantly decreased by 62% and 55%, respectively, compared with the baseline (*p* < 0.001). Five patients (20%) were without levodopa medication 12 months after the operation.

**Conclusions:**

The development of surgical technique based on advanced neuroimaging has improved the outcome of PD STN DBS.

## Introduction

Deep brain stimulation (DBS) of the subthalamic nucleus (STN) has been a surgical treatment option for advanced Parkinson disease (PD) for over two decades in situations where antiparkinsonian medication (APM) is insufficient or poorly tolerated [12, 14]. Its ability to reduce motor symptoms and levodopa-induced dyskinesias has been well-documented [13, 18, 23].

The precise mechanism of STN DBS in Parkinson’s disease is still unknown. It has been shown that stimulation of the dorsolateral part of the STN alleviates most motor symptoms of Parkinson’s disease [21]. It is known that this part of STN is highly cellular with connecting fibers to surrounding structures and the motor cortex, forming the so-called hyperdirect pathway [8]. It also has the highest betaband-activity, the so-called sweet spot [10]. Though the effect of STN DBS with regard to antiparkinsonian medication is well-documented [1], still the combined effect of STN DBS and levodopa and the neural mechanism of action remain unclear [17].

In the early years, DBS surgery was based on constant anatomical coordinates determined by the constant distances from the midcommissural point (MCP) of the anterior commissure–posterior commissure (AC-PC) line, which was measured using intraoperative ventriculography X-rays. This was known as the indirect targeting method [2]. The location of electrodes was controlled also with plain X-ray images in anterior–posterior and lateral projections. The lead’s location accuracy in STN was verified by microelectrode registration (MER) with multiple microelectrodes to enable measuring the electrical activity on a single-neuron level [3, 20]. As imaging technology developed, it became possible to measure MCP from CT or MRI scans [16] and later further development of MRI imaging [4, 9, 15] enabled the possibility of defining patient-specific anatomy of the STN [19] and target direct DBS electrodes. Alongside the development of imaging has enabled precise postoperative analysis of the lead location [5, 7, 11], thus facilitating the optimal programming.

This study was carried out to highlight the clinical and technical development as well as outcome on the basis of single-center experience reflecting a change from an indirect targeting method (iTM) to a direct targeting method (dTM). The aim of this study was to compare the results of the two abovementioned DBS methods used to treat PD patients at Oulu University Hospital, Finland. The hypothesis was that direct targeting of STN (dTM STN DBS) and modern surgical methods improve the efficacy of STN DBS and reduce the need for APM. The outcome was compared with the previous study which was carried out 15 years earlier in the same center with a similar set of PD patients treated using indirectly targeted STN DBS surgery (iTM STN DBS).

## Methods and materials

### The iTM STN DBS study

The previous iTM STN DBS study published in 2006 [6] included PD patients who were operated on from 2001 to 2003 at Oulu University Hospital. Twenty-nine consecutive patients with advanced idiopathic PD underwent primary bilateral STN DBS and had a follow-up at 12 months. In spite of optimal APM, the patients suffered motor fluctuations. The mean preoperative Hoehn and Yahr stage of the patient group was 2.9 (± 0.7 SD).

The STN DBS operations were carried out with Laitinen frame and iTM, which were founded on constant stereotactic coordinates based on information from brain atlases (3 mm posteriorly (*Y*), 5 mm inferiorly (*Z*), and 12 mm lateral (*X*) from the midcommissural point (MCP)) and intraoperative standard ventriculography. Intraoperative stereotactic X-rays in two standard directions (anteroposterior and lateral) were used to control the position of the electrodes. Macrostimulation was carried out intraoperatively (Radionics RFG 5S stimulator, Radionics, Burlington, MA, USA), and after clinical testing, permanent leads were implanted (model 3387, Medtronic, Minneapolis, USA). Perioperative testing was performed using a temporary stimulator (Mattrix, Medtronic, Minneapolis, USA). A permanent implanted pulse generator (IPG) (Kinetra, Medtronic, Minneapolis, USA) was implanted after a successful testing period. Patients were invited for follow-up at 12 months to the same neurosurgical unit, and the clinical outcome was evaluated by the authors. Motor symptoms were evaluated, blinded using Unified Parkinson’s Disease Rating Scale III (UPDRS III). This iTM STN DBS study is described in more detail in the previous publication [6].

### The dTM STN DBS study

Patients included in the dTM STN DBS study were operated on from 2014 to 2017 in the neurosurgical unit of Oulu University Hospital, and all these patients underwent primary bilateral STB DBS surgery. All the patients had advanced, idiopathic PD with motor fluctuations despite optimal APM as assessed by neurologists and no contraindications to DBS treatment. The patients were all evaluated for surgical eligibility and operated by the two neurosurgeons experienced in DBS surgery (ML and MK). The inclusion criteria were as follows: idiopathic PD, at least 5 years from the diagnosis, and no signs of marked decline in cognitive functions or memory. All the patients had a good response to levodopa with at least 30% decrease in motor symptoms tested using UPRDS III. Patient data was collected in a retrospective manner from the DBS protocol of the neurosurgical unit. Ethics Committee and administration of Oulu University Hospital approved the study design. Demographic data for these two patient groups (dTM STN DBS and iTM STN DBS) is shown in Table 1.

**Table 1:** Demographic data for PD patients treated by STN DBS with iTM and dTM

Demographic data	iTM DBS	dTM DBS
Operating years	2001–2003	2014–2017
Number of patients	29	30
Gender (female:male)	9:20	8:22
Excluded number of patients	5	5
Total number of patients	24	25
Age	60 ± 8	61 ± 5
Disease duration (years)	13 ± 7	13 ± 5
Preoperative LED (mg)	585 ± 293	851 ± 368
Preoperative LEDD (mg)	876 ± 473	1158 ± 448
Stereotactic frame	Laitinen	Leksell
Targeting method	Constant coordinates	Direct MRI
Planning image	Intraop ventriculography	Preop 3T DBS-MRI
Intraoperative control imaging	X-ray (AP, lat)	ioCT, O-arm (2D, 3D)
Awake/sleep surgery	Awake	Awake
MER	No	Yes
Macrostimulation	Yes (permanent electrode)	Yes (MER-electrode)
Temporary test–stimulation	Yes	No
Electrode	Medtronic 3387	Medtronic 3389
IPG	Medtronic, Kinetra	Medtronic, Activa PC
Duration on ward (days)	6–17 days (mean 8)	5–10 days (mean 6)
Follow-up (months postop)	12 months	12 months

### Presurgical procedure in the dTM STN DBS study

#### Imaging protocol

Preoperative stereotactic head MRI was done 1–8 weeks before the operation using 3T-MRI with a 32-channel receive-only brain coil (Skyra 3T, Siemens Healthcare GmBH, Erlangen, Germany). All patients were imaged under general anesthesia as outpatients.

The reference dataset used to visualize overall brain structures was sagittal T1-weighted MPRAGE 3D sequence with the contrast agent (repetition time, 2300 ms; echo time, 2.51 ms; flip angle, 8°; averages, 4; field of view, 240 × 240 mm; matric, 352 × 352 px; slice thickness, 0.7 mm; imaging time, 28 min 39 s). Multiple sequences were obtained to demarcate the target region (STN, substantia nigra (SN), zona incerta, posterior limb of capsula interna):

- The coronal (perpendicular to the AC-PC line) T2-weighted spc sequence (repetition time, 1000 ms; echo time, 65 ms; flip angle, 120; averages, 2; field of view, 202 × 202 mm; matrix, 382 × 384 px; slice thickness, 0.5 mm; imaging time, 6 min 21 s)
- The magnitude part of the coronal susceptibility-weighted imaging (SWI) sequence (repetition time, 28 ms; echo time, 20 ms; flip angle, 15; averages, 1; field of view, 200 × 220 mm; matrix, 232 × 256 px; slice thickness, 1.5 mm; imaging time, 5 min 3 s)
- The axial short tau inversion recovery sequence (repetition time, 8000 ms; echo time, 22 ms; inversion time, 120 ms; flip angle, 120; averages, 2; field of view, 235 × 235 mm; matrix, 256 × 256 px; slice thickness, 2.0 mm; imaging time, 13 min 38 s)

For tractography, diffusion tensor images (DTI) were collected from 64 directions (repetition time, 6700 ms; echo time, 109 ms; flip angle, 90; averages, 1; field of view, 260 × 260 mm; matrix, 130 × 130 px; slice thickness, 2.0 mm; imaging time, 7 min 37 s).

#### Software and planning

**Fig. 1:**
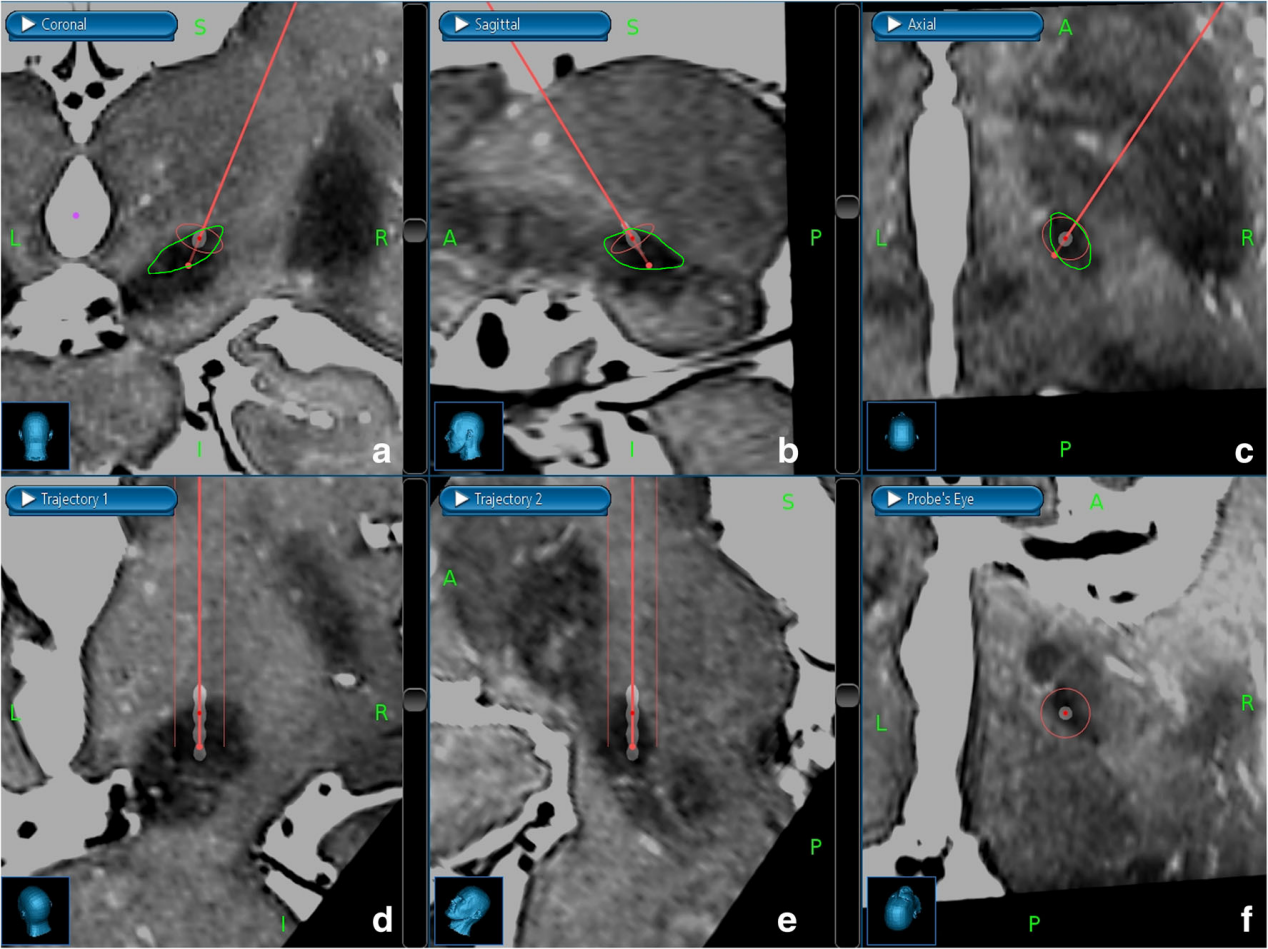
Direct targeting to the dorsolateral border of the subthalamic nucleus (STN, green line) in the right hemisphere. The preoperative stereotactic 3T-MRI T2-sequences and postoperative stereotactic CT scans are fused, and the placement of permanent lead is compared with that of the targeting plan and the location of STN. Upper row: The image fusion is shown in three radiological planes: coronal (**a**), sagittal (**b**), and axial (**c**). The targeting trajectory of the permanent DBS lead (thin red line) is shown with two red dots: the darker red dot (distal) is in the location where the second distal contact of the permanent lead is placed. The lighter red dot (proximal) is in the target point, which is between the most and second proximal contact. Lower row: The image fusion is shown in three planes (subfigures **d**, **e**, and **f**), and the viewing direction is parallel to the targeting trajectory and the permanent lead. All four contacts of the permanent lead are shown. The two middle contacts of the permanent lead are placed into the dorsolateral border of STN. The distal red dot is in the dorsal border, and the proximal red dot is in the ventral border of STN

An operating plan (targeting) was done 1 week prior to surgery using planning software (Stealth FrameLink, Medtronic, Minneapolis, USA) software simply and directly based on 3T-MRI (dTM). The dorsolateral area of the STN was the target. Two middle contacts of the lead were planned for the STN so that the most distal contact was in the SN and the most proximal contact was above the STN (Fig. 1). The trajectory to the target area was planned to avoid vascular structures, ventricles, and the nucleus caudatus (NC). The entry point was planned near the coronal suture.

#### Clinical evaluation

The patients were admitted to the neurosurgical ward 1 day prior to surgery. Preoperative clinical evaluation was made by the first author (ML). Clinical assessment was carried out (UPDRS I–V) without discontinuing the APM during the best medical response, at least 30–60 min after the latest levodopa intake (medON). Documentation was also supplemented by video recordings. All APM was discontinued at least 12 h before surgery.

## Surgical procedure in the dTM STN DBS study

In all cases, the intracranial leads were implanted under local anesthesia and extension wires as well as IPGs were implanted under general anesthesia on the same day.

First, the frame (Leksell G-frame, Elekta, Stockholm, Sweden) was fixed to patient’s head under local anesthesia. The CT coordination indicator box (Leksell, Elekta, Stockholm, Sweden) was attached to the frame and intraoperative stereotactic head CT scans (Toshiba Aquilion One Vision Edition CT-scanner, Canon Medical Systems, Otawara, Tochigi, Japan) were taken. The scanning parameters were 120 kVp, 350 mAs, slice thickness of 0.5 mm, pixel size of 0.48 × 0.48 mm, matrix size of 512 × 512 px, and 320 slices. Contrast enhancement was used to highlight the vascular structures and improve the image fusion. The stereotactic CT scans and preoperative stereotactic 3T-MRI scans were fused in the planning software to obtain stereotactic coordinates *X*, *Y*, and *Z*.

After imaging, the patient was in a supine position on a conventional surgical operating table. The frame was fixed to the table. Next, slightly sedative medication, dexmedetomidine infusion (Dexdor, Orion, Espoo, Finland), was initiated to the patient. Surgical draping was made in a conventional manner.

The stereotactic arc (Leksell Multipurpose Stereotactic Arc, Elekta, Stockholm, Sweden) was attached to the frame; stereotactic coordinates *X*, *Y*, and *Z* were set; and the entry point was located. After administering of local anesthesia, a bifrontal skin incision and burr hole at the entry point were made, starting from the dominant or more symptomatic hemisphere. Administration of sedative medications was stopped at this point. After placing the lower ring of the burr hole cover (StimLoc, Medtronic, Minneapolis, USA, or Guardian, Abbott, IL, USA), a dural incision was made and the stereotactic coordinates were set to the stereotactic arc again. One to three guiding tubes 10 mm before target point (Universal Guide Tube, Elekta, Stockholm, Sweden) were positioned. If the dorsolateral border of the STN was poorly visualized in the stereotactic 3T-MRI scans, the third guiding tube would be placed into the posterolateral position to create a fork-like line penetrating through the dorsolateral border of the STN. One to three microelectrodes (Elekta, Stockholm, Sweden) were inserted through the guiding tubes. Microelectrode recording (MER) was performed (Leadpoint, Alpine Biomed, Skovlunde, Denmark) to evaluate electrical activity from 10 mm above to 2–3 mm below the target point in order to identify the borders of the STN and the electrical firing activity of the STN. Once the boundaries of the STN were determined, three levels were chosen for micromacrostimulation, which was then done using the same MERelectrodes. Stimulation was given with 0 to − 4.0 mA, high frequency 130-Hz current with pulse width 60 μs. Clinical effects and side effects of the stimulation were evaluated and documented by the first (ML) or third author (MK). After the evaluation the location, which gave the strongest STN signal and the best clinical outcome, the microelectrode was replaced with a permanent lead. Quadripolar DBS lead (model 3389, Medtronic, Minneapolis, USA) was used and its two center-most contacts were inserted into the most effective location of the dorsolateral border of the STN. Adjustments of the permanent lead and its depth were made using 2D skull x-rays taken intraoperatively (O-arm, Medtronic, Louisville, CO, USA). The guiding tubes were removed, and a permanent lead was secured in place using the burr hole cover. The distal end of the lead was inserted subcutaneously behind the contralateral ear. The operation was continued repeating the same surgical procedures on the other hemispheres in the same manner. Finally, 3D head CT scanning was done by O-arm to visualize the lead positioning and amount of intracranial air and to rule out intraoperative hemorrhage. This also allowed immediate image fusion with preoperative stereotactic 3T-MRI-scans in order to investigate the lead and contact localization in the STN.

Further, under general anesthesia, extensions (model 37086-40 cm, Medtronic, Minneapolis, USA) and an IPG (Activa PC, Medtronic, Minneapolis, USA) were implanted in the subclavicular region.

All these DBS operations, including MER and clinical testing, were carried out by the two aforementioned neurosurgeons (ML and MK) and one medical physicist (JK).

### Postsurgical procedure in the dTM STN DBS study

A stereotactic head CT was made 1 month postoperatively to ensure that postoperative brain shift and intracranial air were ameliorated, and to exclude postoperative complications such as chronic subdural hematoma. Metal artifact suppression sequences were used to improve the quality of scanning. These new CT images were fused with the preoperative 3T-MRI images, and the final location of the contacts was compared with the preoperative targeting plan (Fig. 1). The contacts with the best location in the STN were identified and taken into account when activating the DBS device.

#### Programming

On the first postoperative day, the stimulation was turned on in a conventional manner using 130 Hz for high-frequency stimulation, 60 μs as pulse width, and 0.5 to 1.0 V as amplitude in both leads. Over the next 3 days, APM was decreased gradually, while the stimulation was increased. One of the two middle contacts of the leads (usually the third contact from the distal end) was activated in a circular fashion according to the information gained from stereotactic CT/3T-MRI fusion.

Further follow-up of the patients took place 1, 3, 6, and 12 months postoperatively for fine adjustments of the DBS programming. The first postoperative control (1 month) was organized overnight in the neurosurgical ward. Further controls were as neurosurgical outpatient visits. The two neurosurgeons and medical physicist responsible for the DBS surgery also made all of the follow-up assessments. After 1 year, the patients returned to their neurologists for the follow-up of PD and DBS with the possibility to consult the neurosurgical DBS unit when needed.

#### Postoperative clinical evaluation

The study end point was evaluated by the first author (ML) 12 months after surgery. Clinical non-blinded assessment was made medON and stimON using UPDRS parts I–V. Clinical status was documented by video recordings.

#### Medication

Data on preoperative APM was recorded from each patient’s history. The medication, which included levodopa, was converted into levodopa equivalencies (LED) by using conversion factors [22]. A total daily levodopa equivalent dose (LEDD) was obtained by calculating together total APM of the day, including dopaminergic and anticholinergic medications and MAO-B inhibitors. Follow-up points for medication assessment were baseline and 1 month, 3 months, 6 months, and 12 months postoperatively, or, as close as possible to that point in time.

### Statistical methods in the dTM STN DBS study

The paired samples *t* test was used for comparisons. Statistical significance was set to *p* < 0.05. All statistical analysis was performed using commercially available software (SPSS for Windows 23.0, IBM, New York, USA).

## Results

Thirty consecutive patients (8 female and 22 male) with idiopathic PD who underwent primary bilateral STN DBS from June 2014 to January 2017 were included in the dTM STN DBS study. One male patient had previously undergone unilateral thalamotomy due to Parkinson tremor, and two patients had previously been treated unsuccessfully with duodenal infusion of levodopa.

Five patients were excluded from the final assessment. One male patient suffered in addition to PD from another neurological disease, which interfered the evaluation of the motor functions, and he was excluded from the study.

Two male patients had an early surgical site infection followed by surgical revision with partial or total DBS removal and cessation of the stimulation. Both of these patients were re-implanted successfully after antibiotic treatment. Two patients, one female and one male, suffered from a technical failure of the DBS device (one lead fracture and one contact damage) followed by cessation of the stimulation or the selection of non-optimal active contact. The patient with a lead fracture was re-implanted successfully but 12-month evaluation was exceeded. The patient with the contact damage got a satisfactory response to treatment by altering the programming. All these complications were treatable by conventional manners causing no permanent morbidity or mortality in our study population.

A total of 25 patients (7 female and 18 male) were included in the analysis. Three out of these 25 patients, had postoperative bleeding complications. One male patient, who suffered from postoperative unilateral chronic subdural hematoma, which did not require any surgical intervention nor affect the clinical outcome, was included in the analysis. Two patients, female and male, suffered from postoperative acute hemorrhage alongside one or both electrodes. For the female patient, the unilateral hemorrhage caused a slight transient confusion which did not affect the clinical assessment and the patient was included in the final results. The male patient with bilateral hemorrhage had a subclinical vitamin K deficiency diagnosed after postoperative evaluation. Due to ongoing rehabilitation, the patient was lost from follow-up and the 12-month clinical follow-up evaluation (UPDRS) was not available and only medication data was included. However, all three of these patients made a full recovery.

The stimulation of STN was overall well-tolerated among the patients. There were no significant speech problems due to stimulation. One male patient had slight irritability in his personality which ameliorated after the change from monopolar to bipolar stimulation.

The mean age of the patients at the time of the operation (STN DBS) was 61 years ± 5 (mean ± SD) and the mean disease duration (time from the PD diagnose to surgery) was 13 ± 5 years.

MER was performed on all patients on both hemispheres. In 22 patients out of 25, MER was started from the left hemisphere. From these cases, three microelectrodes were used in 13 patients, two in 11 patients, and one in one patient. The micro- and micromacrostimulation influenced electrode positioning in the left hemisphere in eight cases (32%) out of 25. The direction was from central to anteromedial.

In the right hemisphere, three microelectrodes were used in 11 patients, two in 13, and one in one patient. The micro- and micromacrostimulation influenced electrode positioning in the right hemisphere in 11 cases (44%), and the direction was also from central to anterior or anteromedial.

**Fig. 2:**
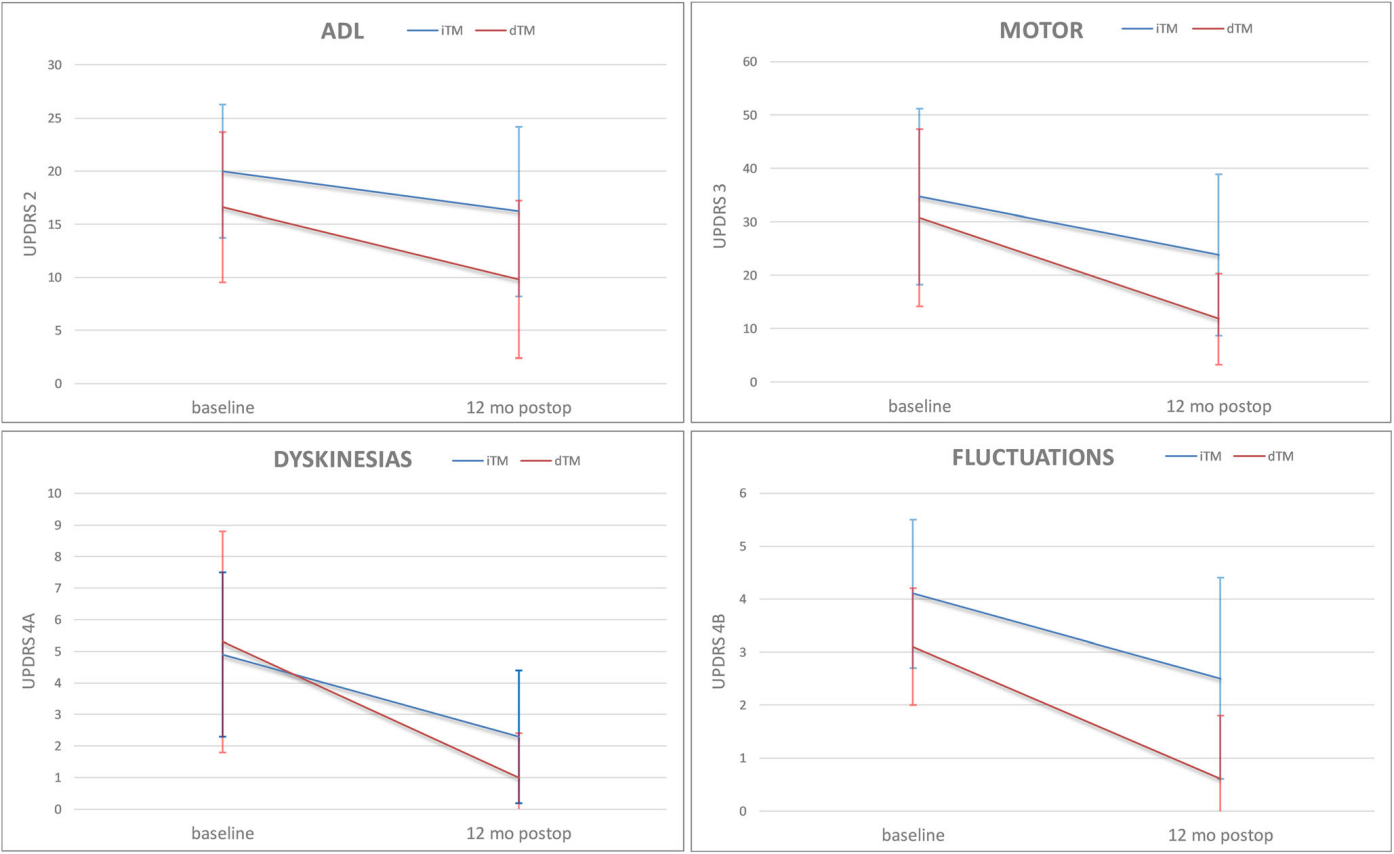
Unified Parkinson’s Disease Rating Scale (UPDRS) scores of the patients with Parkinson’s disease (PD) treated by STN DBS with two different targeting methods: indirect (iTM, blue line) and direct (dTM, red line). After the 12-month follow-up with medication and stimulation on, the difference was highly significant (*p* < 0.001) in four subscores of UPDRS: the activities of daily living (ADL), motor, dyskinesias, and fluctuations

Twelve months after the STN DBS operation, significant improvement was seen in six out of seven parameters of the UPDRS during medON and stimON (Table 2). The change was statistically highly significant (*p* < 0.001) in four parameters: activities of daily living (ADL, UPDRS II), motor score (UPDRS III), dyskinesias (UPDRS IVa), and fluctuations (UPDRS IVb). The ADL and motor score improved by 41% and 62%, respectively, after the 12-month follow-up. Dyskinesias and fluctuations were both reduced by 81% after the 12-month follow-up (Table 2, Fig. 2). The reduction of motor subscores was also statistically highly significant in two areas: rigidity and akinesia. Rigidity was reduced by 74% and akinesia by 66% due to stimulation (Table 3).

**Table 2:** UPDRS scores of PD patients treated by STN DBS with two different targeting methods: iTM DBS (24 PD patients, indirect targeting method with constant coordinates) and dTM DBS (25 PD patients, direct targeting method with 3T-MRI)

Parameter	UPDRS part	UPDRS no.	Max. value	Before surgery medON			12 months after surgery medON stimON		
				iTM DBS	dTM DBS	p*	iTM DBS	dTM DBS	p**
M, B, and M	I	1–4	16	3.6 ± 2.2	1.8 ± 1.8	0.003	3.1 ± 2.7	2.2 ± 2.1	0.198
ADL	II	5–17	52	20.0 ± 6.3	16.6 ± 7.1	0.083	16.2 ± 8.0^3^	9.8 ± 7.4^1^	0.006
Motor	III	18–31	108	34.7 ± 16.5	30.8 ± 16.6	0.414	23.8 ± 15.1^3^	11.8 ± 8.5^1^	0.001^1^
Dyskinesias	IVa	32–35	13	4.9 ± 2.6	5.3 ± 3.5	0.653	2.3 ± 2.1^2^	1.0 ± 1.4^1^	0.014
Fluctuations	IVb	36–39	7	4.1 ± 1.4	3.1 ± 1.1	0.008	2.5 ± 1.9^2^	0.6 ± 1.2^1^	0.000^1^
Complications	IVc	40–42	3	0.9 ± 0.9	1.7 ± 1.0	0.005	0.5 ± 0.6	0.9 ± 0.9^2^	0.075
H and Y	V	43	5	2.9 ± 0.7	2.7 ± 0.7	0.323	2.6 ± 0.9	2.2 ± 0.7^2^	0.088

M, B, and M = mentation, behavior, and mood

H and Y = Hoehn and Yahr

^1^*p* ≤ 0.001

^2^*p* < 0.01

^3^*p* < 0.05

*Preoperative *p* value between iTM DBS patients and dTM DBS patients (*t* test)

**Postoperative (12 months) *p* value between iTM DBS patients and dTM DBS patients (*t* test)

**Table 3:** UPDRS motor subscores of STN DBS patients operated on by two different targeting methods: iTM DBS (24 PD patients, targeted indirect method with constant coordinates) and dTM DBS (25 PD patients, targeted direct method with 3T-MRI), on medication

Score	UPDRS no.	Max.	Before surgery			12 months after surgery medON stimON		
			iTM DBS	dTM DBS	p*	iTM DBS	dTM DBS	p**
Speech	18	4	2.0 ± 0.8	1.0 ± 0.9	0.000	1.7 ± 1.1	0.8 ± 0.8	0.002
Tremor	20–21	28	5.0 ± 5.5	3.1 ± 4.1	0.176	2.1 ± 3.2	1.1 ± 0.2	0.126
Rigidity	22	20	6.6 ± 5.1	6.6 ± 1.0	1.000	4.1 ± 3.9	1.7 ± 0.3^1^	0.004
Akinesia	23–26	32	13.1 ± 6.5	14 ± 1.4	0.502	9.2 ± 5.4	4.8 ± 0.8^1^	0.000
Gait	29	4	1.0 ± 0.6	0.8 ± 0.2	1.121	1.0 ± 0.8	0.4 ± 0.2	0.000
Post. stabil.	30	4	1.0 ± 1.0	0.5 ± 0.2	0.018	0.9 ± 0.9	0.4 ± 0.1	0.008

^1^*p* < 0.001

*Preoperative *p* value of motor subscores between iTM DBS patients and dTM DBS patients (*t* test)

**Postoperative (12 months) *p* value of motor subscores between iTM DBS patients and dTM DBS patients (*t* test)

**Fig. 3:**
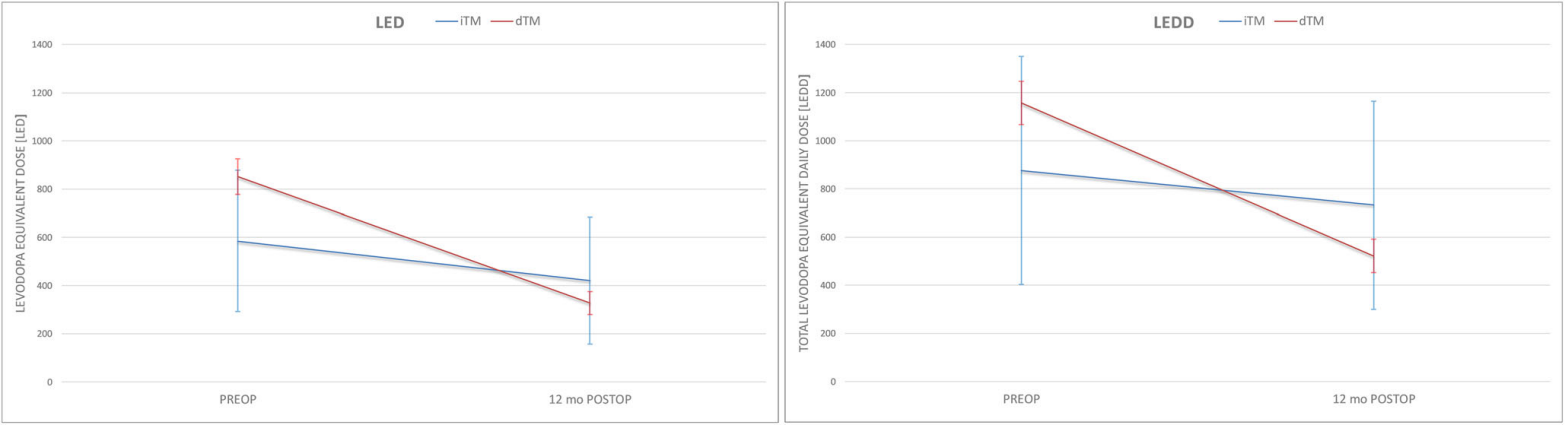
Reduction of the antiparkinsonian medication (levodopa equivalent dose, LED, and total levodopa equivalent daily dose, LEDD) in PD patients treated by STN DBS using two different targeting methods: indirect (iTM) and direct (dTM). Baseline and after the12-month follow-up

Alongside adjustments to the stimulator parameter, antiparkinsonian medication was reduced, mainly during the first postoperative week. After 12-month follow-up, LED and LEDD were significantly lower, 62% and 57%, respectively, compared with baseline (Fig. 3). Both reductions were statistically highly significant (*p* < 0.001). For seventeen out of twenty-five patients, the reduction of LED was over 50%, and for nine patients, it was over 75% compared with the baseline. Five patients (20%) were without levodopa medication 12 months after the operation; their preoperative LEDs were 500, 333, 466, 600, and 665 mg (mean 513 mg ± 114 SD). Three patients (12%) were without any antiparkinsonian medication 12 months after the operation; their preoperative LEDDs were 586, 740, and 865 mg (mean 730 mg ± 114 SD) (Table 4).

**Table 4:** Antiparkinsonian medication of STN DBS patients operated on by two different targeting methods: iTM DBS (24 PD patients, targeted indirect method with constant coordinates) and dTM DBS (25 PD patients, targeted direct method with 3T-MRI)

	Preoperative			12 months postoperative		
	iTM DBS	dTM DBS	p*	iTM DBS	dTM DBS	p**
LED	585 ± 293	851 ± 74	0.000	421 ± 264^1^	327 ± 48^1^	0.086
LEDD	876 ± 473	1158 ± 90	0.005	732 ± 432^2^	522 ± 70^1^	0.021

LED = levodopa equivalent dose (mg)

LEDD = total daily levodopa equivalent dose (mg)

^1^*p* < 0.001

^2^*p* = 0.003

*Preoperative *p* value of LED between iTM DBS patients and dTM DBS patients (*t* test)

**Postoperative (12 months) *p* value of LEDD between iTM DBS patients and dTM DBS patients (*t* test)

Twelve months after each operation, the mean parameters of stimulation were amplitude 2.4 ± 0.5 V, pulse width 71 ± 19 μs, and frequency 142 ± 25 Hz. Twenty-one patients had bilateral monopolar stimulation, three patients had bilateral bipolar stimulation, and one patient had unilateral monopolar and unilateral bipolar stimulation. Eleven patients had two contacts activated, and one patient had three contacts activated in the left hemisphere’s lead. Eight patients had two active contacts, and one had three active contacts in the right hemisphere’s lead.

The mean duration of the postoperative hospitalization was 6 days. A prolonged stay in the neurosurgical ward after the operation was mainly due to complications after surgery or long traveling distances in the Northern Finland.

## Discussion

This study confirms that STN DBS in advanced PD is an effective treatment to substantially reduce motor symptoms and complications associated with levodopa medication. This study provides updated information concerning STN DBS in PD patients in Northern Finland, and describes the use of the direct targeting method and MER in DBS-surgery as well as standardized and predictable programming method in detail. This study also highlights the progress that has been made in DBS surgery over the past years by comparing the results of this study with the previous one [6].

The results of this study show that in the dTM STN DBS group, the motor outcome improved significantly in four subscores of the UPDRS: ADL, motor scores, dyskinesias, and fluctuations when measured with medication and stimulation on. These dTM STN DBS findings can be compared with the findings of the previous iTM STN DBS study [6] in the same center 15 years earlier. The results in dTM STN DBS were better than in iTM STN DBS: ADL 41% vs. 19%, motor score 62% vs. 31%, dyskinesias 81% vs. 53%, and fluctuations 81% vs. 39% (Fig. 2). The difference between motor and fluctuation scores 12 months after the operation is statistically highly significant (Table 2). This could be explained by the difference in targeting method and MER used. In the earlier study, the DBS operation and lead location were planned with an indirect targeting method based on standard coordinates from brain atlases. The intraoperative imaging was limited to ventriculography x-ray images, and the permanent lead location was confirmed using macrostimulation. In the present study, the DBS operation was planned individually based on the particular patient’s brain anatomy available from high-quality 3T-MRI information and was confirmed using MER, micromacrostimulation, and 3D intraoperative O-arm imaging [23]. However, it is noteworthy that MER and micromacrostimulation were used for finetuning the location of permanent leads in the left and the right hemispheres (32% and 44%, respectively) and thus affect the outcome.

Postoperative evaluation of the leads and contact location with MRI/CT fusion images provide the information necessary for more predictable adjustments of the stimulation parameters instead of depending on trial and error. This advancement became possible due to the technical progress of imaging technologies and their insightful introduction to DBS surgery.

The direct targeting method in STN DBS results in a greater reduction in both levodopa medication (LED) and total antiparkinsonian medication (LEDD) when compared with the indirect targeting method (62% vs. 28% and 55% vs. 16%, respectively) (Fig. 3). It should be noted that in preoperative medication (LED, LEDD), there is a clear statistical difference between these two patient groups (*p* = 0.000 and *p* = 0.005, respectively) (Table 4). In the dTM STM DBS group, patients had a higher preoperative dose of antiparkinsonian medication without increases of dyskinesias. This was probably due to the introduction of combination drugs, for example, dopamine agonists and entacapone. It is notable that in the dTM STM DBS group, 20% of the PD patients were without levodopa medication and 12% did not have any antiparkinsonian medication 12 months after the operation. Previous studies have shown that this reduction in antiparkinsonian medication can be long-lasting [1].

It is also noteworthy that in the dTM STN DBS group, the reduction of antiparkinsonian medication did not negatively impact motor response (UPDRS III) 12 months after the operation. In the motor subscores, rigidity, and akinesia in particular, the improvement was notable. However, it should be noted that 12 months after operation, the motor part of UPDRS (III) was performed blinded in the earlier study and non-blinded in the recent study. Stimulation parameters in the dTM STN DBS group were quite equal compared with the iTM STN DBS group. However, the latter had a wider range of parameters which demonstrates the heterogeneity of programming parameters: amplitude 2.4 V ± 0.5 SD, pulse width 71 μs ± 19 SD, and frequency 142 Hz ± 25 SD vs. amplitude 2.7 V ± 1.1 SD, pulse width 77 μs ± 16 SD, and frequency 171 Hz ± 13 SD, respectively. The heterogeneity may be due to inaccurate positioning of the electrodes in the STN region and the slight variations of normal brain structures. In addition, it should be noted that the permanent electrodes used were slightly different. Medtronic leads 3387 and 3389 have the same length of contacts (1.5 mm) but the distance of the contacts differs (1.5 mm and 0.5 mm, respectively). However, in conventional stimulation through one active contact, the stimulation field is the same for both 3387 and 3389 electrodes. The limitations of this study are a relatively small but rather homogenous and well-characterized patient sample and its non-randomized nature. However, current knowledge implies that it would be unethical to carry out a double-blinded, randomized study in order to compare these two targeting methods. Noteworthy, the Laitinen frame is no longer in clinical use. In about one-third of cases, MER and intraoperative test stimulation finetuned the location of the permanent lead. This has undeniably influenced the clinical outcome in addition to MRI targeting. MER in this kind of form is probably unnecessary in modern DBS surgery.

Due to the previous study design, clinical assessment was performed without discontinuing APM; however, this limitation does not influence the outcome in ADL and APM. The difference between these two patient groups is noticeable in preoperative UPDRS parts I (mentation, behavior, mood), IVb (fluctuations), and IVc (complications) where the difference is statistically significant. However, in the most important parameters of this study preoperative UPDRS II (ADL), III (motor), and IVa (dyskinesias), the difference between these two groups is not statistically significant. In this regard, the patient groups can be expected to represent similar patient groups. The postoperative clinical assessment (UPDRS) was performed blinded in the earlier study and non-blinded in the latter, and this can affect the outcome. Nevertheless, this study demonstrates how progress in surgical and imaging technology has improved the outcome for PD patients undergoing STN DSB surgery.

## Conclusions

STN DBS is an effective surgical method that decreases motor symptoms and levodopa-derived fluctuations in advanced, idiopathic PD. Direct 3T-MRI-based DBS targeting combined with MER and micromacrostimulation improves the outcome significantly at the 12-month follow-up and decreases the need for antiparkinsonian medication compared with the baseline and the previous results of the era of indirect targeting. High-accuracy surgical MR imaging and direct targeting methods are essential to improving the results of STN DBS. Postoperative imaging and greater knowledge and understanding of the location of the leads and contacts improve and simplify the programming of the DBS device. DBS patients benefit when DBS surgery and follow-up are carried out at the same DBS center. Further studies are needed to explore long-term results in a larger PD population with STN DBS and more precise targeting based on the neuronal activity and connections of STN with the assistance of intraoperative microelectrode recording (MER) and diffusion tensor imaging (DTI).

## Abbreviations

AC-PC: Anterior commissure–posterior commissure
APM: Antiparkinsonian medication
DBS: Deep brain stimulation
DTI: Diffusion tensor images
dTM: Direct targeting method
IPG: Implanted pulse generator
iTM: Indirect targeting method
LED: Levodopa equivalent
LEDD: Total daily levodopa equivalent daily dose of antiparkinsonian medication
MCP: Midcommissural point
medOFF: Not medicated
medON: Medicated
MER: Microelectrode registration
PD: Parkinson’s disease
STN: Subthalamic nucleus
STN DBS: Deep brain stimulation in the subthalamic nucleus
UPDRS: I–V Unified Parkinson’s Disease Rating Scale parts 1–5
